# Day and Night: Circadian Rhythms in Worms

**DOI:** 10.1371/journal.pbio.1000511

**Published:** 2010-10-12

**Authors:** Rachel Jones

**Affiliations:** Freelance Science Writer and Editor, Welwyn, Hertfordshire, United Kingdom

**Figure pbio-1000511-g001:**
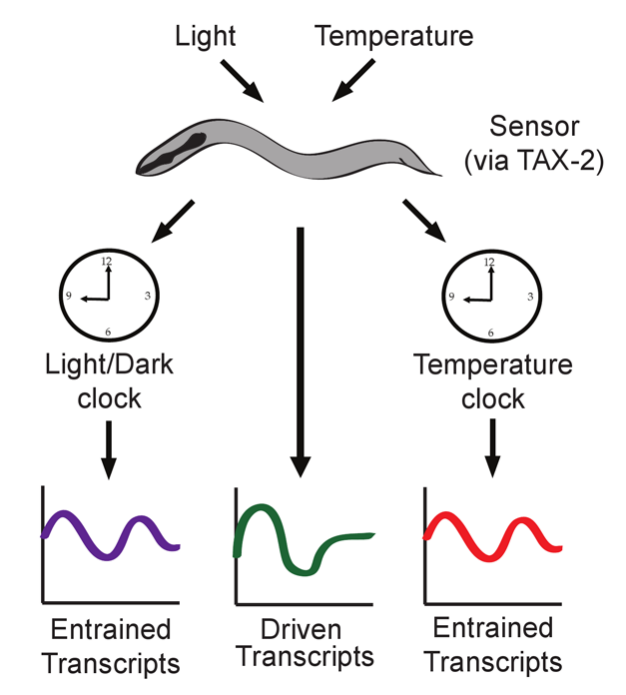
Light and temperature cycles both drive and entrain rhythmic gene expression in *C. elegans*.

[Fig pbio-1000511-g001]Most organisms seem to show “circadian rhythms”—cycles of behaviour or gene expression that repeat roughly every 24 hours. Such cycles represent an internal circadian clock that can be “entrained,” or synchronized, to a regular light/dark or temperature cycle, and will then continue to cycle in the absence of the light or temperature stimulus.

In the nematode worm *Caenorhabditis elegans*, however, it has been hard to identify definite circadian rhythms. Although behavioural cycles have been described, these are unusually variable. And gene expression oscillations on a 24-hour cycle, such as those found in many other animals, have not been observed—until now. In a new study published in this issue of *PLoS Biology*, van der Linden et al. used a technique called genome-wide expression profiling to identify genes whose transcripts were rhythmically expressed and exhibited characteristics of being under circadian control.

The authors used growth-synchronized populations of worms (in which all the worms are the same age), which were kept under a 12-hour/12-hour cycle of either light/dark or warm/cold from an early larval stage until adulthood (the entrainment period). By collecting RNA from the adult worms every four hours, they could look for transcripts that were expressed more or less at a particular point in the 24-hour cycle. The authors also then placed the worm populations in “free-running” conditions—constant darkness (for the light/dark entrained animals) or a constant temperature (for the warm/cold entrained animals)—and again took samples of RNA every four hours, to look for transcripts that were expressed cyclically even in the absence of an external stimulus.

The analysis identified three categories of genes with cyclical transcription. First, stimulus-driven genes were transcribed in a circadian fashion under a light/dark or temperature cycle, but not under free-running conditions, showing that they were not controlled by an endogenous clock. Second, genes that appeared to be under circadian control showed cyclical transcription both during entrainment and under constant conditions, when no external driver for the rhythm existed. And third, some genes showed constant transcription during the entrainment period but cyclical transcription under free-running conditions, suggesting that they are controlled by a circadian clock, but that their cyclical expression is somehow masked under a light/dark or temperature cycle.

Interestingly, the genes whose transcription was entrained by the light/dark cycle were generally different from those entrained by the temperature cycle. Although diverse biological processes were represented in both sets of genes, certain functions were more highly represented in one or the other group; for example, genes that control reproductive development and locomotion were more likely to be entrained by the temperature cycle than the light/dark cycle. In addition, more genes were entrained by temperature as by light.

A number of genes, such as *per* (*period*) and *tim* (*timeless*), are known as “clock genes” because they have been implicated in the regulation of circadian rhythms in various species, including *Drosophila* and mammals. However, the homologs of these genes in *C. elegans* did not show rhythmic transcription in these experiments, which suggests that they might not act as clock genes in worms. Alternatively, they could show cyclical expression only in a few cells, which this kind of whole-organism analysis might not detect, or their protein levels could show cyclical expression.

To look more closely at circadian gene expression in *C. elegans*, the authors selected a particular temperature-entrained gene (the neuropeptide gene *nlp-36*) and made a “tagged” gene construct. Animals bearing this construct would express green fluorescent protein (GFP) wherever *nlp-36* is normally expressed, and this can be visually assessed. The expression of GFP could be seen to oscillate under the temperature entrainment conditions, and continued to do so for at least two days at constant temperature, although the size of the oscillation decreased.

How do external stimuli—light or temperature—entrain the expression of circadian genes? In *C. elegans*, a gene called *tax-2*, which encodes a cyclic nucleotide-gated channel, is required for responses to both light and temperature. van der Linden et al. found that the entrainment of *nlp-36* expression by temperature was impaired in *tax-2* mutant worms, as was the entrainment by light of three genes that oscillated under the light/dark cycle in normal worms. This implies that sensory transduction mediated in part by *tax-2* is required for the entrainment process both in light/dark and temperature cycles.

*C. elegans* is an important genetic model organism for many areas of research. A fuller understanding of circadian rhythm cycles in this species is of interest not only to researchers looking at internal clocks; it will also provide insight into how the behaviour and physiology of *C. elegans* is modulated by circadian cycles, which might have implications for many areas of research that use these worms as a model.

**van der Linden AM, Beverly M, Kadener S, Rodriguez J, Wasserman S, et al. (2010) Genome-Wide Analysis of Light and Temperature-Entrained Circadian Transcripts in *C. elegans*. doi: 10.1371/journal.pbio.1000503**

